# Genetic predisposition to elevated total immunoglobulin E levels defines a distinct adult-onset-predominant asthma phenotype

**DOI:** 10.1038/s41598-026-37679-5

**Published:** 2026-01-29

**Authors:** Takashi Matsuda, Hironori Masuko, Yu Ozawa, Rie Shigemasa, Haruna Kitazawa, Yohei Yatagai, Nobuyuki Hizawa

**Affiliations:** 1 Department of Respiratory Medicine, Institute of Medicine, University of Tsukuba, Ibaraki, Japan; 2 Department of Respiratory Medicine, Kasumigaura Medical Center, Ibaraki, Japan

**Keywords:** Asthma, Immunoglobulin E, Genome-wide association study, IgE polygenic risk scores, Diseases, Genetics, Immunology, Medical research

## Abstract

Asthma heterogeneity remains a major barrier in precision medicine. Although elevated total serum immunoglobulin E (IgE) is a hallmark of asthma, even in nonatopic patients, its causal role in asthma pathogenesis is debated. We hypothesized that genetic predisposition to increased IgE defines a distinct asthma endotype. A genome-wide association study of total serum IgE in 1,287 non-asthmatic Japanese adults was used to construct IgE polygenic risk scores (IgE_PRS). Applying IgE_PRS to 745 patients with asthma, we performed cluster analysis incorporating age at onset, total IgE levels, IgE_PRS, and percent predicted forced expiratory volume in 1 s (pFEV_1_), identifying four distinct adult asthma phenotypes. Notably, one cluster had the highest IgE_PRS and adult-onset-predominant type 2 inflammation. Conversely, the second cluster displayed the highest IgE levels but average IgE_PRS. The remaining two clusters comprised patients with lower IgE_PRS. One cluster was characterized by eosinophilia and smoking-related airflow limitation, whereas the other exhibited a type 2 low phenotype. In a 10-year retrospective cohort, over 30% of newly diagnosed asthma cases fell into the genetically predisposed high-IgE_PRS cluster. These findings reveal a distinct adult-onset-predominant asthma phenotype driven by genetically determined IgE production, offering new avenues for endotype-driven diagnosis and personalized therapy.

**Supplementary Information:**

The online version contains supplementary material available at 10.1038/s41598-026-37679-5.

## Introduction

 Asthma is a heterogeneous disease, with clinical and molecular diversity that challenges conventional classification and management^1^. Traditionally, asthma has been divided into T2-high and T2-low subtypes based on the presence of type 2 inflammation; however, emerging evidence suggests that this dichotomy does not fully capture the complexity of asthma pathogenesis. Although T2-high asthma is characterized by eosinophilic inflammation and elevated levels of type 2 cytokines, such as interleukin (IL)-4, IL-5, and IL-13, which are often associated with allergen sensitization, recent studies have indicated that total serum immunoglobulin E (IgE) levels can be elevated even in the absence of atopy^2^, reflecting distinct pathogenic mechanisms. Furthermore, both genetic and environmental factors, including viral infections, smoking, and air pollution, can induce airway inflammation by disrupting the epithelial barrier and modulating networks of cytokines, such as thymic stromal lymphopoietin (TSLP), IL-33, and IL-25^3^.

Although IgE production is typically induced by allergen stimulation^4^, IgE exerts important effects on mast cells, even in the absence of allergens. Specifically, IgE stabilizes high-affinity immunoglobulin E receptor (FcεRI) on the cell surface and prevents its internalization, thereby enhancing FcεRI expression^5^. Furthermore, IgE binding to FcεRI inhibits apoptosis and promotes mast cell proliferation^6^. In addition, IgE engagement can induce a conformational shift of FcεRI from a dimeric to a monomeric form, which amplifies immune signaling and contributes to mast cell survival, migration, and differentiation^7^. Therefore, we hypothesize that there is a group of patients with type 2 asthma whose pathogenesis is driven by genetic susceptibility to elevated total IgE levels independent of allergen sensitization.

Total IgE levels exhibit substantial inter-individual variability and are influenced not only by environmental factors, but also, to a significant extent, by genetic predisposition^8–10^. Several genome-wide association studies (GWASs) have been conducted to investigate the genetic architecture of total serum IgE levels. In studies of European populations, several loci, including *FCER1A*, *STAT6*, *IL13*, and the major histocompatibility complex region, are significantly associated with total IgE levels^11^. Our group has previously reported a significant association between a single nucleotide polymorphism (SNP) in the human leukocyte antigen (*HLA*)*-C* region and total serum IgE levels^12^. We also identified a polymorphism in the *ORMDL3/GSDMB* locus, which was previously associated with childhood-onset asthma, as a variant related to total IgE levels; however, this SNP showed no significant association with allergen sensitization^13^. Although comparison between genetic factors associated with total IgE levels and those associated with asthma have revealed some shared susceptibility loci, large-scale GWASs have indicated limited overlap between total IgE-associated loci and asthma risk loci, suggesting that elevated total IgE levels may not be a direct causal factor for asthma pathogenesis^14^.

Therefore, in this study, we aimed to elucidate how genetic predisposition to elevated total IgE levels contributes to the heterogeneity of asthma, with a particular focus on adult-onset-predominant phenotypes. To this end, we investigated the association between distinct asthma phenotypes and a polygenic risk score (PRS) reflecting genetic susceptibility to high total IgE levels, calculated from GWAS-identified SNPs in a Japanese population.

## Results

### Immunoglobulin E (IgE) polygenic risk score (PRS) in non-asthmatic controls

**Fig. 1:**
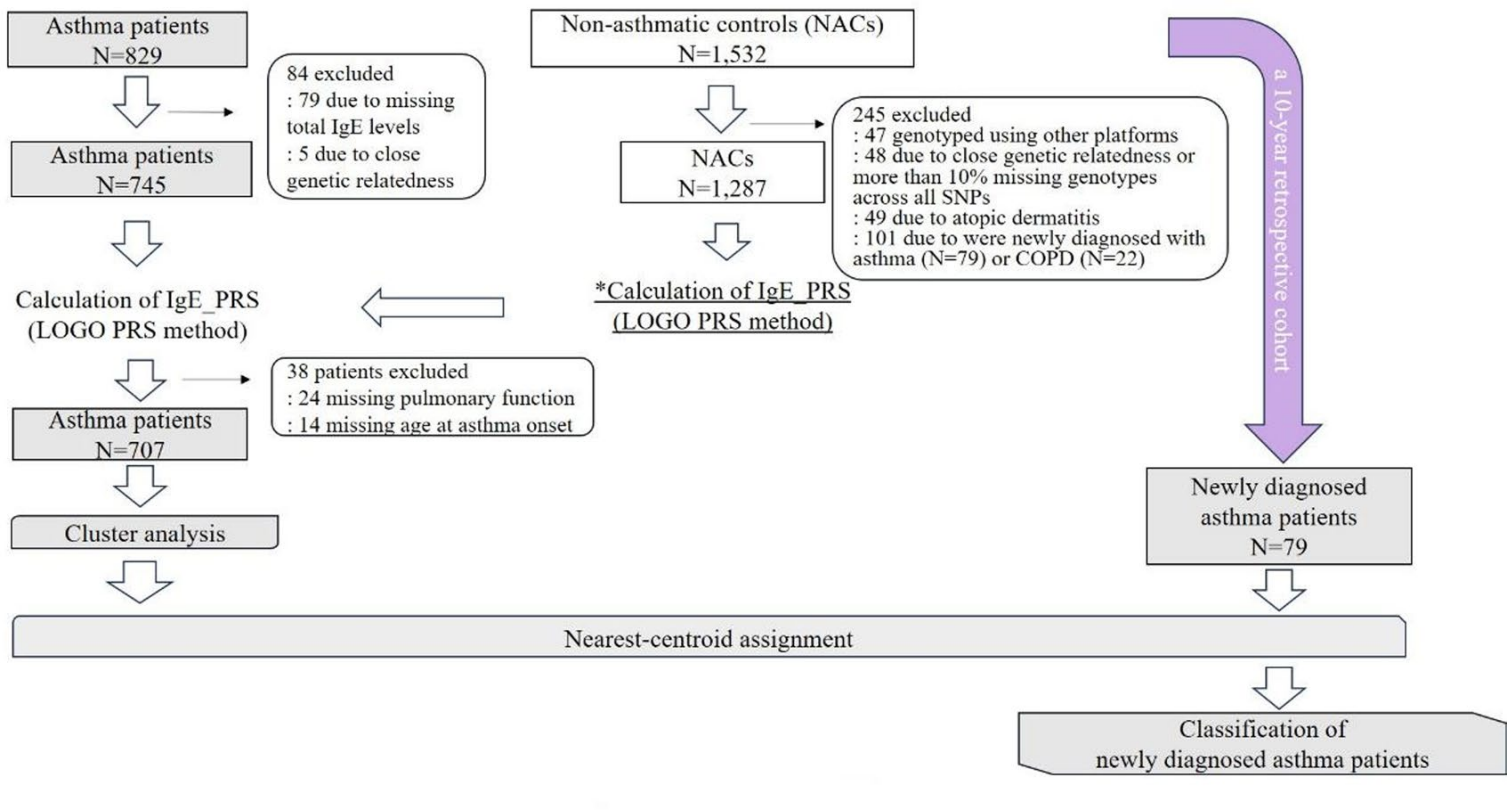
Study flow diagram.

The overall study design is shown in Fig. 1. Among the 1,532 non-asthmatic controls, 47 participants were genotyped using other platforms, 49 with atopic dermatitis, and 101 with newly developed asthma (*n* = 79) or chronic obstructive pulmonary disease (COPD) (*n* = 22) during the 10-year observation period were excluded. Additionally, 48 participants were excluded based on genetic quality control criteria: three individuals with a genotype missing rate > 10%, 31 with heterozygosity rates ± 3 standard deviation (SD) from the mean, and 14 identified as first- or second-degree relatives were excluded based on genotyping data. Consequently, 1,287 non-asthmatic controls were included in the population for IgE_PRS calculation. Of these, 662 (51.4%) were female participants. The mean age was 51.3 (range, 30–84) years, and the mean body mass index (BMI) was 23.0 kg/m^2^ (SD, 3.1). A total of 718 (55.8%) participants were never-smokers, and 745 (57.8%) were sensitized to at least one aeroallergen. The mean level of total IgE levels was 1.78 log_10_ IU/mL (SD, 0.58) (Table 1).

We used a longitudinal cohort of 1,532 individuals without asthma at baseline, wherein asthma development was retrospectively assessed over a 10-year follow-up period. Among them, 47 participants were genotyped using other platforms, 49 with atopic dermatitis, and 101 who developed asthma (*n* = 79) or chronic obstructive pulmonary disease (*n* = 22) during follow-up were excluded. Forty-eight participants were excluded due to either close genetic relatedness or > 10% missing genotypes across all single nucleotide polymorphisms. The remaining 1,287 non-asthmatic controls were used for calculating immunoglobulin E polygenic risk score (IgE_PRS) using the leave-one-group-out PRS method. Of the 829 patients with asthma, 79 and five were excluded due to missing total IgE data and close genetic relatedness, respectively. Among the 745 patients with asthma, the IgE_PRS was similarly calculated for non-asthmatic controls. After excluding 38 participants with missing data on pulmonary function or age at asthma onset, cluster analysis was performed.

**Table 1:** Characteristics of the participants.

	Non-asthmatic controls	Patients with asthma	P*
Number of participants	1287	745	
Sex (female, %)	662 (51.4)	427 (57.3)	0.010
Age, y (range)	51.3 (30–84)	58.3 (19–90)	4.2 × 10^− 24^
Age at asthma onset (range)		41.6 (0–87)	
BMI (SD)	23.0 (3.1)	23.6 (4.0)	6.6 × 10^− 4^
Smoking pack-years (%)			0.014
0	718 (55.8)	442 (60.5)	
0–10	168 (13.0)	106 (14.5)	
> 10	399 (31.0)	182 (24.9)	
Atopy** (%)	745 (57.8)	498 (70.4)	3.1 × 10^− 8^
Total IgE levels (log, SD)	1.78 (0.58)	2.21 (0.62)	1.8 × 10^− 44^
IgE_PRS Z score (SD)	0.00 (1.00)	0.048 (0.78)	0.073
pFEV_1_ (%, SD)	92.4 (12.5)	83.8 (23.4)	5.8 × 10^− 19^
FEV_1_ /FVC (%, SD)	82.6 (5.7)	70.9 (12.7)	7.5 × 10^− 95^

*Categorical variables were compared using the chi-square test, while continuous variables were compared using the t-test. Linear regression analysis was performed with sex, age, BMI, smoking pack-years, and atopy as covariates for total IgE levels and IgE_PRS Z score.

**Atopy was defined as the presence of allergic sensitization for at least one inhaled allergen.

Missing data among non-asthmatic controls included smoking pack-years (*n* = 2), pFEV₁ (*n* = 3), and FEV₁/FVC (*n* = 3). Among the patients with asthma, missing data included age of asthma onset (*n* = 14), BMI (*n* = 36), smoking pack-years (*n* = 15), atopy (*n* = 38), pFEV₁ (*n* = 24) and FEV₁/FVC (*n* = 13).

BMI, body mass index; IgE, immunoglobulin E; IgE_PRS, immunoglobulin E polygenic risk score; FEV₁, forced expiratory volume in 1; FVC; forced vital capacity; pFEV₁, predicted forced expiratory volume in 1 s; SD, standard deviation.

**Fig. 2:**
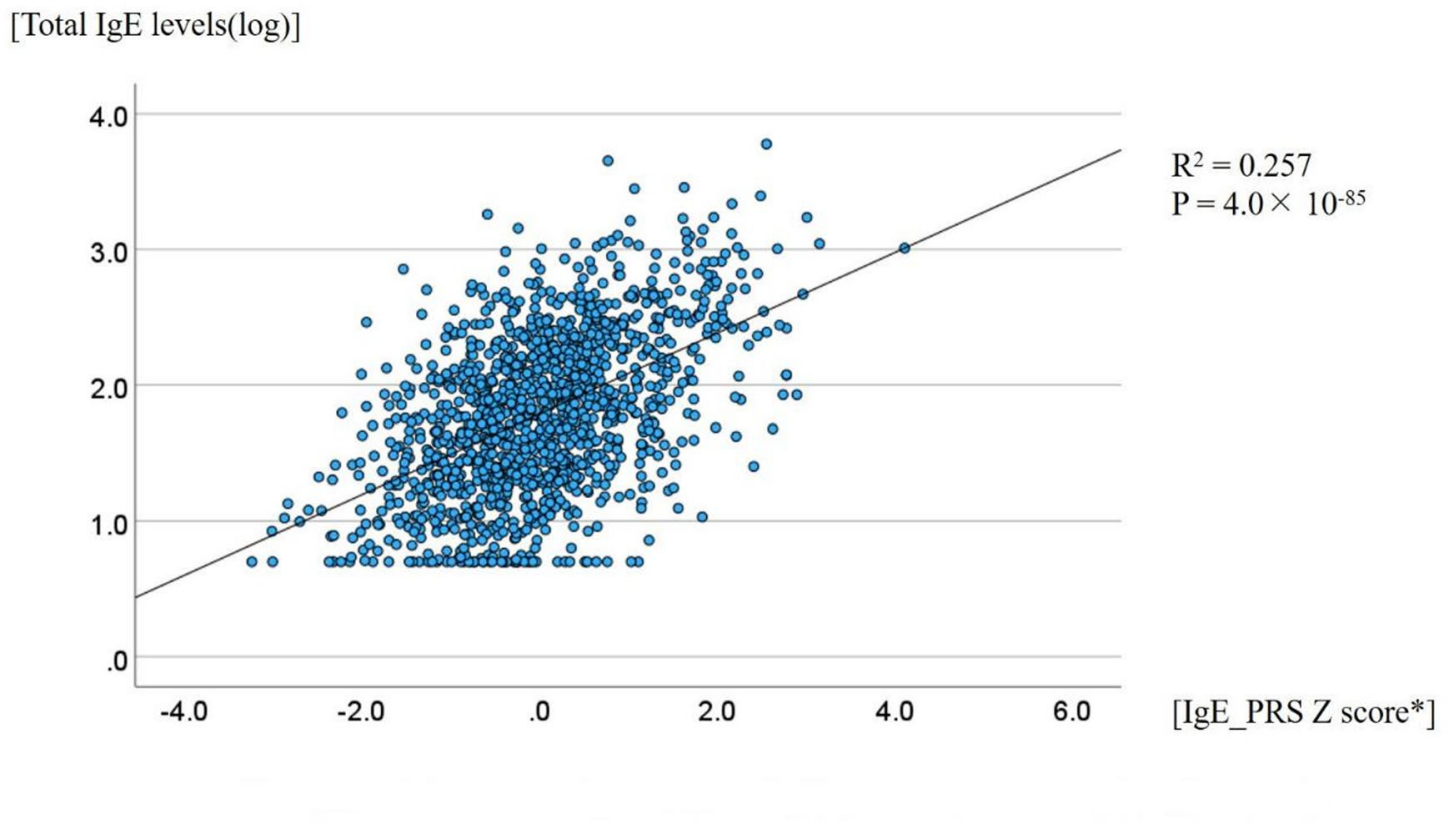
Correlation between total immunoglobulin E (IgE) levels and IgE_polygenic risk score (PRS) in 1287 non-asthmatic controls.

As illustrated in Supplementary Figure S1, the IgE_PRS was constructed using the leave-one-group-out (LOGO) cross-validation method. The 1,287 non-asthmatic controls were randomly divided into five subsets, and five PRSs were generated. These scores were meta-analyzed using the inverse-variance weighting method. The resulting IgE_PRS, as a surrogate for genetic predisposition to elevated total IgE levels, showed a statistically significant correlation with total IgE levels (Fig. 2, R^2^ = 0.257, *P* = 4.0 × 10⁻⁸⁵). This is of a similar magnitude of correlation with that reported in previous studies^15,16^; however, methodological differences should be noted. In terms of clinical features, elevated levels of total IgE levels were significantly associated with male sex (*p* = 0.0049), smoking status (*p* = 1.8 × 10⁻⁵), and allergic sensitization (*p* = 2.5 × 10⁻⁵⁰) (Supplementary Figure S2). In contrast, IgE_PRS was not significantly associated with any clinical characteristics (Supplementary Figure S3). These findings support the use of IgE_PRS as a valid instrumental variable using Mendelian randomization (MR) analysis, meeting the two core assumptions of relevance to exposure and independence from confounding variables^17^.

Among 1,287 non-asthmatic controls, using Pearson’s correlation analysis, a positive association between total IgE levels and the IgE_PRS Z score was found (R^2^ = 0.257, *p =* 4.0 × 10^–85^).

The IgE_PRS Z score was calculated as follows: Z = (X − µ)/σ, where X is the individual IgE_PRS and µ and σ represent the mean and standard deviation of IgE_PRS among the 1,287 non-asthmatic controls.

The figure was created using IBM SPSS Statistics version 29.0.0.0.

### Comparison of total IgE levels, IgE_PRS and other clinical parameters between non-asthmatic controls and patients with asthma

**Fig. 3:**
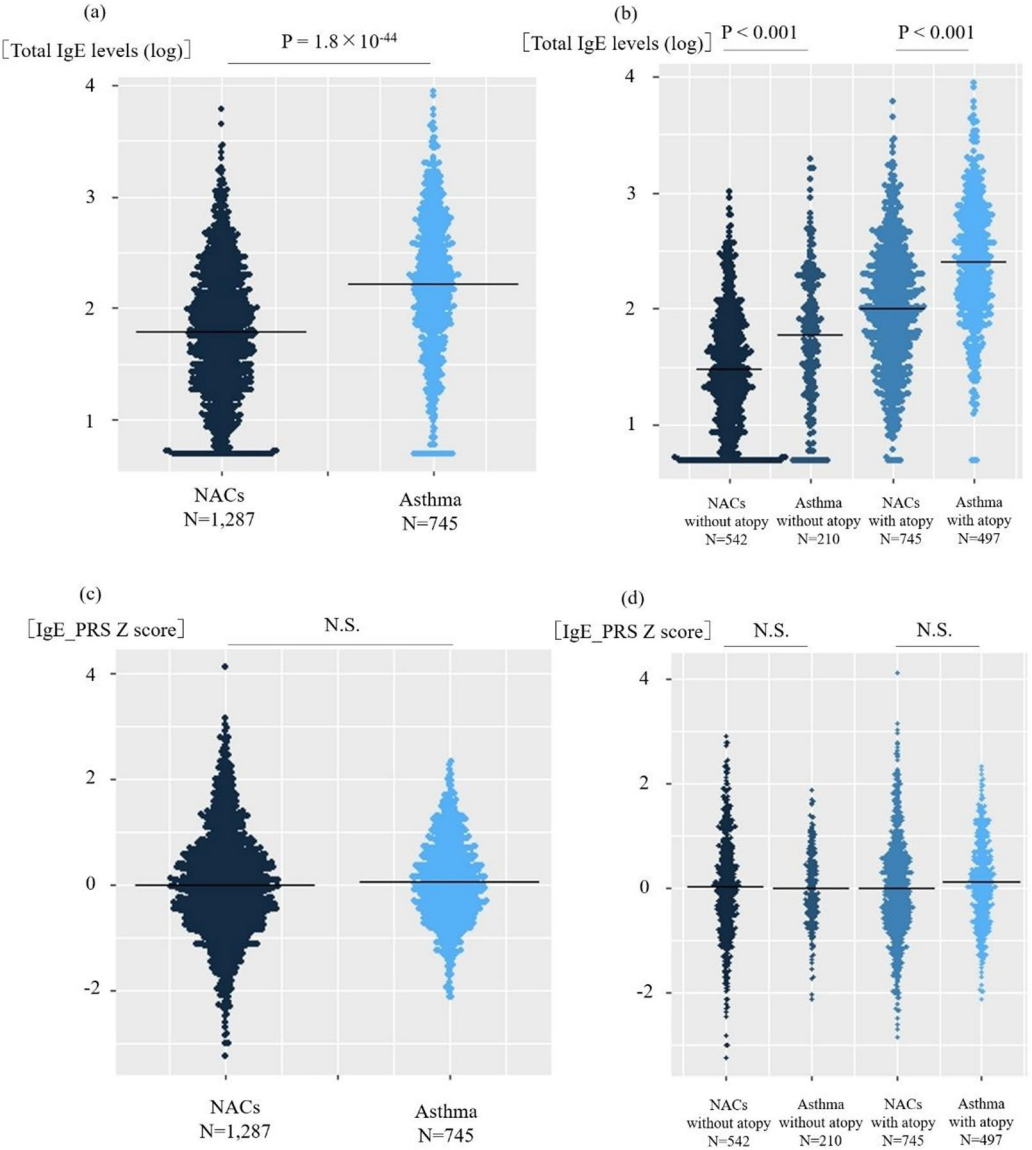
Comparison of total immunoglobulin E (IgE) levels and IgE_polygenic risk score (PRS) between non-asthmatic controls and patients with asthma. (**a**) Beeswarm plot of total IgE levels between non-asthmatic controls (NACs) and patients with asthma. Linear regression analysis was performed with sex, age, BMI, smoking status, and atopy as covariates. Atopy was defined as the presence of allergic sensitization for at least one inhaled allergen. (**b**) Beeswarm plot of total IgE levels among NACs and patients with asthma stratified based on atopy status. Using the Kruskal–Wallis test, significant differences among the groups were found (*p* < 1.0 × 10^− 15^), followed by Dunn-Bonferroni-corrected pairwise comparisons. (**c**) Beeswarm plot of IgE_PRS between the NACs and patients with asthma. Linear regression was created using the same covariates as in (**a**). (**d**) Beeswarm plot of IgE_PRS among the NACs and patients with asthma with or without atopy. The Kruskal–Wallis test and Dunn-Bonferroni tests were performed: no significant differences were found. The figure was generated using R version 4.3.2. BMI, body mass index.

In the asthma cohort, 79 patients with missing total IgE levels and 5 showing high genetic relatedness were excluded; consequently, data of 745 patients were analyzed. The IgE_PRS calculation formula constructed using the LOGO approach in non-asthmatic individuals was also applied to patients with asthma. Total IgE levels were significantly higher in patients with asthma compared with non-asthmatic controls (*p* = 1.8 × 10^− 44,^ Fig. 3a). When we stratified the participants according to the presence of allergic sensitization status, total IgE levels remained significantly higher in patients with asthma than in non-asthmatic controls in both non-sensitized and sensitized subgroups (both, *p* < 0.001; Fig. 3b). In contrast, no significant difference in IgE_PRS was observed between non-asthmatic controls and patients with asthma (Fig. 3c). Similarly, stratified analysis showed no differences in IgE_PRS across the non-sensitized and sensitized subgroups (Fig. 3d).

Other clinical characteristics were also compared between non-asthmatic controls and patients with asthma (Table 1). Patients with asthma showed female predominance (*p* = 0.010), had slightly higher BMI (*p* = 6.6 × 10^− 4^), and showed a higher prevalence of allergic sensitization (*p* = 3.1 × 10^− 8^). In addition, they exhibited lower pFEV₁ (*p* = 5.8 × 10^− 19^) and FEV₁/FVC (*p* = 7.5 × 10^− 95^) compared with non-asthmatic controls.

### Identification of an asthma cluster with high IgE_PRS

In patients with asthma, cluster analysis was performed using IgE_PRS, total IgE level, age at asthma onset, and pFEV₁ as input variables. The number of clusters was determined using the Bayesian Information Criterion (BIC), which showed a marked reduction up to four clusters and a plateau thereafter. In addition, the silhouette index for the four-cluster solution was approximately 0.3, indicating a stable and acceptable clustering structure. Thus, the optimal number of clusters was automatically determined to be four (Supplementary Figure S4).

**Fig. 4:**
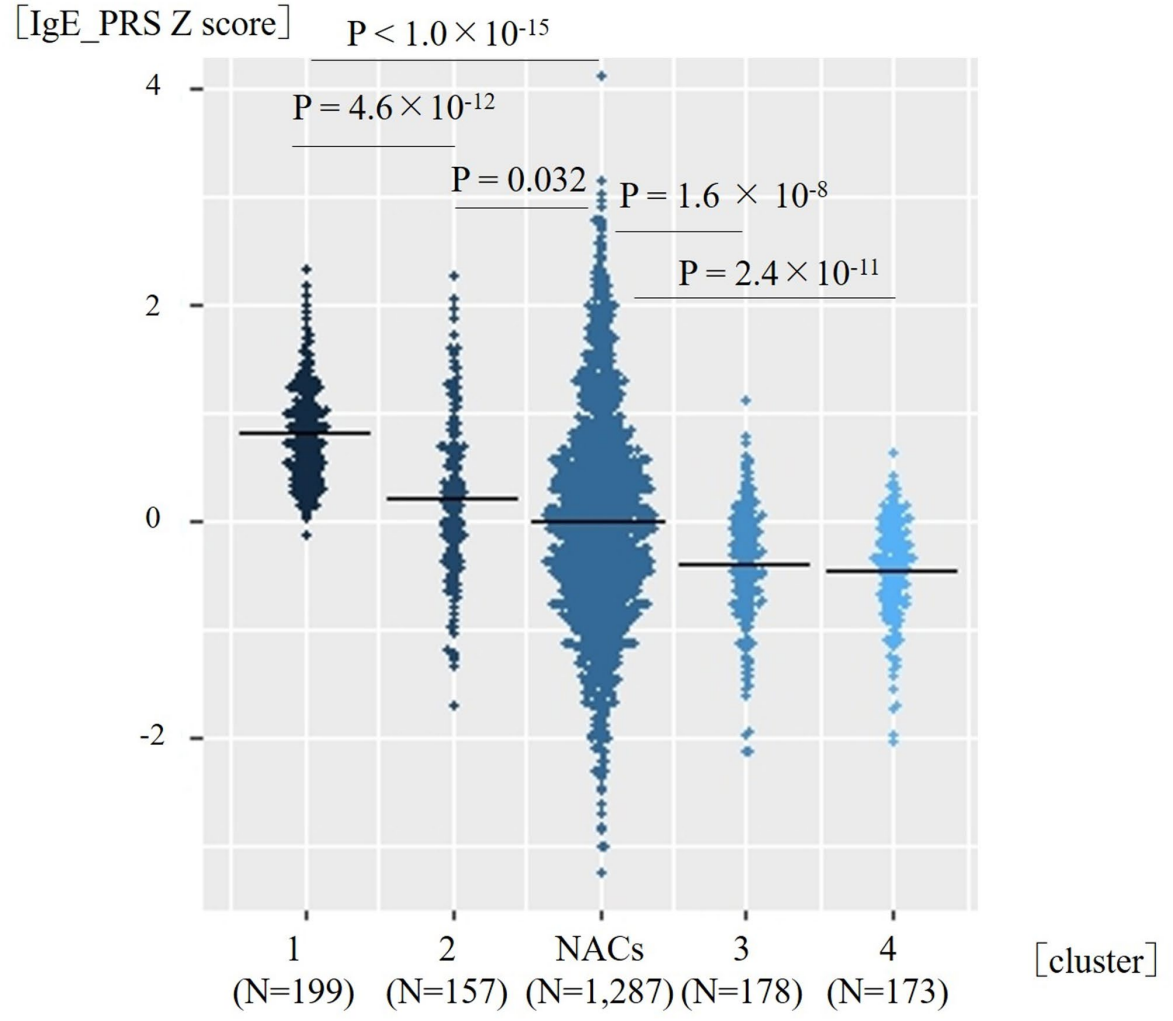
Beeswarm plots of immunoglobulin E (IgE)_polygenic risk score (PRS) across the asthma clusters and non-asthmatic controls.

Clusters 1–4 were labeled in descending order of mean IgE_PRS. Compared with non-asthmatic controls, both Clusters 1 and 2 showed significantly higher IgE_PRS than did non-asthmatic controls (corrected *p* < 1.0 × 10⁻^15^ and corrected *p* = 0.032, respectively); Moreover, Cluster 1 showed the highest IgE_PRS, exceeding that of Cluster 2 (corrected *p* = 4.6 × 10⁻^12^). Whereas Clusters 3 and 4 showed significantly lower IgE_PRS than did non-asthmatic controls (corrected *p* = 1.6 × 10⁻^8^ and corrected *p* = 2.4 × 10⁻^11^, respectively) (Fig. 4; Supplementary Table S1 and S2).

Using the Kruskal–Wallis test, significant differences among the groups (*p* < 1.0 × 10^− 15^) were found, followed by using Dunn-Bonferroni-corrected pairwise comparisons. Both Clusters 1 and 2 showed significantly higher IgE_PRS than did non-asthmatic controls; Cluster 1 showed the highest IgE_PRS, exceeding that of Cluster 2 and all other clusters. The figures were generated using R version 4.3.2.

**Fig. 5:**
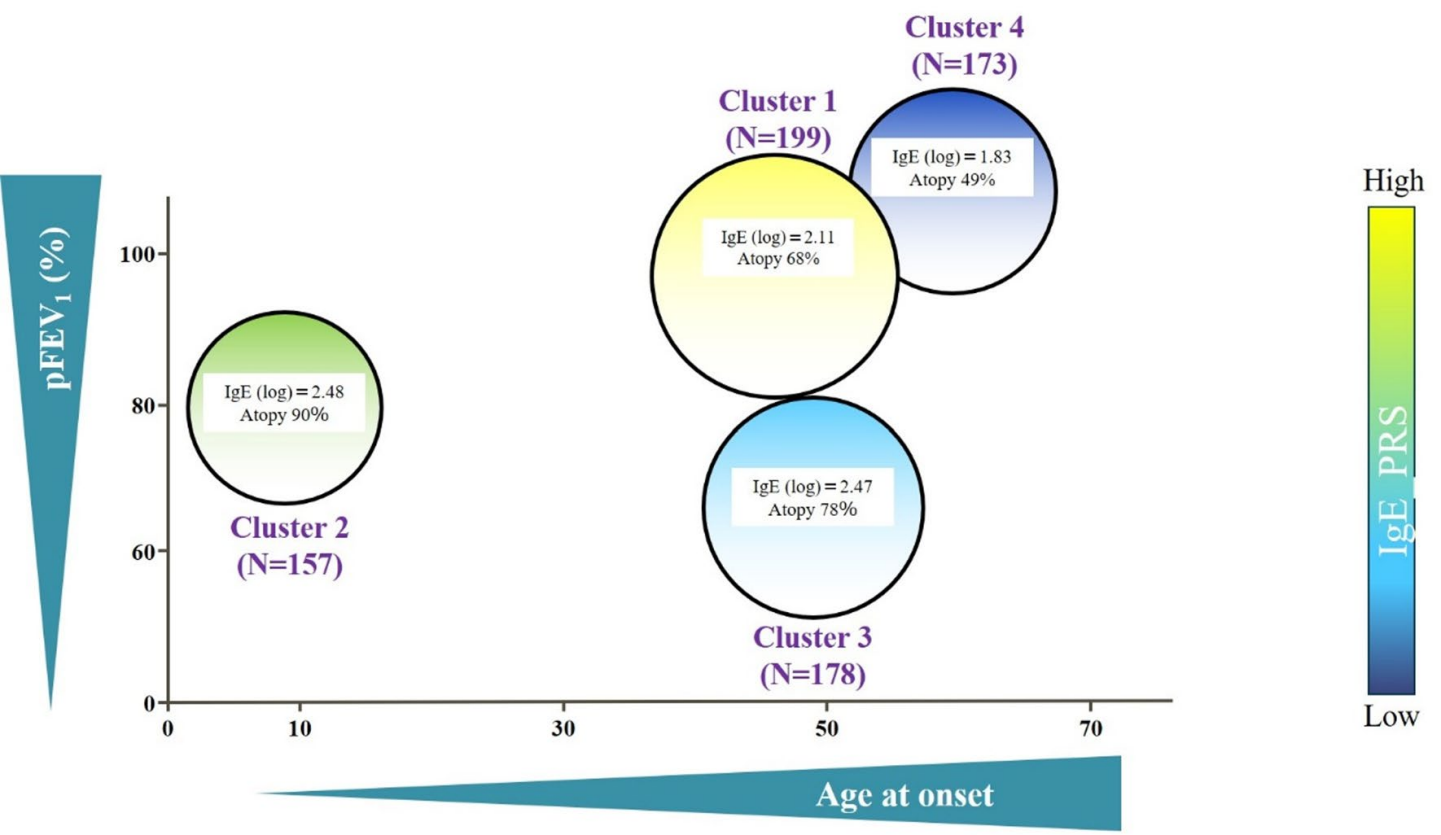
Conceptual illustration of the asthma clusters. The adult asthma clusters are characterized by differences in immunoglobulin E (IgE)_polygenic risk score (PRS), total IgE levels, age at asthma onset, pFEV_1_, and the prevalence of atopy. Atopy was defined as allergic sensitization to at least one inhaled allergen. Cluster 1 (C1) had the highest IgE_PRS levels, whereas Cluster 4 (C4) had the lowest. Distinct clinical and immunological profiles were observed across the clusters. pFEV₁, predicted forced expiratory volume in 1 s.

Table 2 summarizes the characteristics of the adult patients with asthma in each cluster. A conceptual illustration of cluster distribution based on IgE_PRS, age at asthma onset, and pFEV₁ is provided in Fig. 5. Cluster sizes were as follows: Cluster 1 (*n* = 199), Cluster 2 (*n* = 157), Cluster 3 (*n* = 178), and Cluster 4 (*n* = 173). Cluster 1, which exhibited the highest IgE_PRS, had the third-highest total IgE level (mean, 2.11 log_10_ IU/mL). The mean age at asthma onset was 48.9 years, and the mean pFEV₁ was 87.1%. This cluster had the second-highest proportion of female participants (*n* = 125, 62.8%) and an intermediate prevalence of allergic sensitization (*n* = 134, 68.0%). Cluster 2, with a moderate IgE_PRS, had the highest total IgE level among all clusters. The mean age at asthma onset was 9.9 years, and the mean pFEV₁ was 79.7%. This cluster exhibited the highest prevalence of allergic sensitization (*n* = 133, 89.8%) and included the largest number of individuals with comorbid atopic dermatitis (*n* = 34, 26.1%). Cluster 3, which ranked third in terms of IgE_PRS, had the second-highest total IgE level. The mean age at asthma onset was 49.2 years, and the mean pFEV₁ was 64.7%. Allergic sensitization was present in 133 (78.6%) participants, and this cluster had the highest proportion of current or former smokers (*n* = 98, 55.6%). Cluster 4, which was characterized by the lowest IgE_PRS, also had the lowest total IgE levels. The mean age at asthma onset was 54.8 years, which was the oldest among all clusters. Allergic sensitization was present in 78 (48.7%) participants, and this cluster had the highest proportion of female participants (*n* = 114, 65.8%).

Significant differences in sex, smoking status, atopy prevalence, and comorbid atopic dermatitis among the four clusters were found, as determined by the chi-squared tests (*p* = 3.5 × 10^− 4^, 3.3 × 10^− 7^, 5.0 × 10^− 15^, and 1.9 × 10^− 15^, respectively). Regarding continuous variables, the Kruskal–Wallis test was performed (Table 2 and Supplementary Table S3).

Based on Dunn-Bonferroni post-hoc analysis, regarding asthma at onset, patients in Cluster 2 were younger than those in Cluster 1, Cluster 3, or Cluster 4 (all corrected *p* < 1.0 × 10^− 15^), whereas Clusters 1 and 3 did not significantly differ. Patients in Cluster 4 were older than those in Clusters 1 and 3 (corrected *p* = 0.012 and 0.0099, respectively). Patients in Cluster 2 had the highest total IgE levels, higher compared with Cluster 1 (corrected *p* = 6.0 × 10⁻^8^) but not significantly different from Cluster 3. Patients in Cluster 1 showed the third-highest total IgE levels, which were higher compared with Cluster 4 (corrected *p* = 5.9 × 10^− 4^). BMI did not differ across clusters. Although blood eosinophil counts did not significantly differ among the four clusters, they tended to be higher in Clusters 2 and 3. The mean pFEV₁ decreased progressively from Cluster 4 to 1, 2, and 3, with all pairwise differences being statistically significant (corrected *p* = 1.1 × 10^− 11^, 0.025 and 1.9 × 10^− 9^, respectively).

**Table 2:** Characteristics of the four asthma clusters.

	Cluster 1	Cluster 2	Cluster 3	Cluster 4	P*
Number of participants	199	157	178	173	
Sex (female, %)	125 (62.8)	82 (52.2)	82 (46.0)	114 (65.8)	3.5 × 10^− 4^
Age, y (range)	61.0 (23–84)	44.0 (19–86)	63.5 (26–90)	63.6 (21–89)	< 1.0 × 10^− 15^
Age at asthma onset (range)	48.9 (5–82)	9.9 (0–33)	49.2 (21–83)	54.8 (20–87)	< 1.0 × 10^− 15^
BMI (SD)	23.8 (4.0)	23.5 (4.0)	23.6 (4.5)	23.5 (3.8)	0.681
Smoking pack-years (%)					3.3 × 10^− 7^
0	124 (62.6)	101 (65.1)	78 (44.3)	123 (71.5)	
0–10	22 (11.1)	29 (18.7)	30 (17.0)	22 (12.7)	
> 10	52 (26.2)	25 (16.1)	68 (38.6)	27 (15.6)	
Atopy (%)	134 (68.0)	133 (89.8)	133 (78.6)	78 (48.7)	5.0 × 10^− 15^
Atopic dermatitis (%)	4 (2.5)	34 (26.1)	7 (5.2)	2 (1.4)	1.9 × 10^− 15^
Allergic rhinitis (%)	47 (23.6)	50 ^a^ (31.8)	36 (20.2)	37 (21.3)	0.111
Total IgE levels (log, SD)	2.11 (0.54)	2.48 (0.52)	2.47 (0.60)	1.83 (0.61)	< 1.0 × 10^− 15^
Blood eosinophil count (/µl)	303.2 (282.1)	366.9 (364.0)	356.7 (353.8)	275.7 (264.8)	0.096
pFEV_1_ (%, SD)	87.1 (19.9)	79.7 (20.7)	64.7 (17.0)	103.8 (17.1)	< 1.0 × 10^− 15^
FEV_1_/FVC (%, SD)	72.3 (11.3)	72.5 (12.4)	63.5 (13.5)	76.5 (8.9)	< 1.0 × 10^− 15^

*Statistical comparisons were performed between the four asthma clusters. Categorical variables were compared using the chi-squared test. Continuous variables were compared using the Kruskal-Wallis test. Blood eosinophil counts were log-transformed and analyzed using the Kruskal-Wallis test. The results of post hoc comparisons are detailed in Supplementary Table S3.

Missing data across the four asthma clusters were:

BMI: Two, 12, five, and four patients in clusters 1, 2, 3, and 4, respectively.

Smoking pack-years: one, two, two, and one patient in clusters 1, 2, 3, and 4, respectively.

Atopy: Two, nine, nine, and 13 patients in clusters 1, 2, 3, and 4, respectively.

Atopic dermatitis: 39, 27, 43, and 34 patients in clusters 1, 2, 3, and 4, respectively.

Allergic rhinitis: 39, 27, 43, and 33 patients in clusters 1, 2, 3, and 4, respectively.

Blood eosinophil count: 39, 24, 35, and 29 patients in clusters 1, 2, 3, and 4, respectively.

BMI, body mass index; IgE, immunoglobulin E; FEV₁, forced expiratory volume in 1; FVC; forced vital capacity; pFEV₁, predicted forced expiratory volume in 1 s; SD, standard deviation.

### Gene prioritization based on GWASs of cluster 1

To further characterize the genetic background of Cluster 1, we performed two genome-wide association analyses. First, GWAS comparing patients in Cluster 1 with non-asthmatic controls revealed 52 mapped genes (Supplementary Table S4), many of which were located in the *HLA* region, including *HLA-C* and other class I and class II genes. Second, GWAS comparing Cluster 1 with the combined group of Clusters 2–4 indicated 29 mapped genes (Supplementary Table S5), again showing enrichment of loci in the *HLA* region. In addition to classical *HLA* class I and class II genes, several other mapped genes not encoding MHC molecules were also identified, including genes related to immune regulation and epithelial or barrier-associated processes (e.g., *HCG22*, *FLOT1*,* TCF19*,* CDSN*, and *DDR1*), as well as innate immune–related genes such as *MICA*,* MICB*, and *C4A*.

### A 10-year retrospective observational cohort study on asthma development

Among the 1,532 non-asthmatic individuals in the retrospective cohort, 79 newly developed asthma during the 10-year observation period. Among them, using the nearest-centroid assignment model, 25 cases (31.6%) were classified into Cluster 1, eight (10.1%) into Cluster 3, and 46 (58.2%) into Cluster 4 (Supplementary Table S6).

## Discussion

In this study, we aimed to clarify whether genetic predisposition to elevated total IgE levels defines novel asthma endotypes, particularly adult-onset-predominant asthma. We addressed this by constructing a PRS for total serum IgE based on GWAS data in non-asthmatic Japanese adults and examined its relationship with the clinical phenotypes of adult asthma. Initially, we demonstrated that total serum IgE levels were significantly higher in patients with asthma than in non-asthmatic controls, even after adjusting for sex, age, smoking status, and allergic sensitization. However, no difference in IgE_PRS (a surrogate for genetic predisposition to increased total IgE levels, but not to allergic sensitization) was observed between the overall asthma cohort and non-asthmatic controls. Therefore, elevated total IgE levels do not universally contribute to asthma pathogenesis. Rather, given the heterogeneity of asthma, they may exert a causal influence in certain subgroups, independent of known atopic and demographic factors.

We identified Cluster 1 as having a unique phenotype with the highest IgE_PRS (Fig. 4). In addition, in a 10-year retrospective observation period of non-asthmatic controls, Cluster 1 accounted for approximately 30% of the newly diagnosed patients with asthma, suggesting that the specific asthma phenotype driven by genetic susceptibility to increased IgE is prevalent in adult-onset asthma cases. This cluster may be driven by a strong genetic predisposition to elevated total IgE levels and was clinically characterized by adult-onset-predominant asthma, mild airflow limitation, increased levels of total serum IgE, and other features consistent with a T2-high phenotype (Table 2; Fig. 5).

To better understand the pathogenesis of Cluster 1, we performed two GWASs : one comparing Cluster 1 with non-asthmatic controls and the other comparing Cluster 1 with the combined group of Clusters 2–4 (Supplementary Table S4 and S5). In addition to the GWAS that compared Cluster 1 with non-asthmatic controls, the strongest and most consistent association signal was located within the *HLA* locus (6p21.3) when GWAS compared asthma patients within Clusters 1 and 2–4. Therefore, variations within the *HLA* region may significantly contribute to the genetic structure of Cluster 1 along with asthma susceptibility genes. Our previous GWAS of total IgE levels identified variants in the *HLA-C* region in a Japanese population^12^. The observed association between total serum IgE levels and genetic variants in the *HLA* region may reflect an intrinsic predisposition to enhanced IgE production, independent of antigen-specific immune responses^12,18^.

In addition, the *HLA* region encompasses numerous immune regulatory genes that collectively shape the local cytokine milieu and influence B-cell activation. Among the genes associated with Cluster 1 (Supplementary Table S4 and S5), several are of particular interest in the context of antigen-independent IgE hyperresponsiveness. For instance, *MICA* and *MICB* encode stress-induced ligands that activate innate immune cells, contributing to a baseline pro-inflammatory state that may facilitate nonspecific IgE synthesis^19^. Complement components, such as *C4A*, are positively correlated with elevated IgE levels and blood eosinophil counts^20^, suggesting that aberrant complement activation may promote IgE hyperresponsiveness via innate immune mechanisms. *HCG22* has been reported to be associated with late-onset adult asthma^21^, HIV disease progression and HIV-1 C acquisition^22^, as well as total white blood cell counts^23^, suggesting a role for *HCG22* in immune regulation. Several other non-*MHC* genes identified in this region may further contribute to the heterogeneity of epithelial barrier abnormalities and altered innate and adaptive immune responses in this subgroup. These genes include *FLOT1*,* and TCF19* (immune regulation)^24,25^, as well as *CDSN* and *DDR1* (epithelial and barrier-related processes)^26,27^.

Cluster 2 was characterized by moderately elevated IgE_PRS, higher than those in non-asthmatic controls but second only to Cluster 1, which was associated with childhood-onset-predominant asthma, the highest levels of total serum IgE, and the highest prevalence of allergic sensitization and atopic dermatitis. These features are consistent with the concept of “atopic march,” which posits that impaired skin barrier function in infancy can lead to the sequential development of atopic dermatitis, followed by allergic airway disease in later life^28^. Moreover, genetic susceptibility to rhinovirus infection is associated with the development of allergic asthma during childhood^29^. Disruption of the airway barrier due to viral infection can exacerbate type 2 inflammation and promote allergen sensitization. Therefore, this mechanism is presumed to contribute to the pathogenesis of this cluster.

Despite elevated total IgE levels and frequent allergic sensitization, Cluster 3 exhibited a low IgE_PRS. A previous study identified a severe asthma phenotype with similar characteristics, including late-onset and increased eosinophil counts, and overlap with COPD^30^. Given that a Japanese study on smoking-related asthma identified T2-high and T2-low subtypes in patients with severe asthma^31^, Cluster 3 may correspond to the T2-high subtype, in which asthma is primarily driven by smoking-related mechanisms. Although this group does not have a genetic predisposition to elevated IgE, total IgE levels remained high, possibly reflecting environmentally induced innate immune activation, such as increased TSLP^32^. In contrast, patients in Cluster 4 exhibited features of non-type 2 asthma. This cluster was characterized by the oldest age at onset, lowest IgE_PRS, lowest total serum IgE concentration, lowest eosinophil count, and lowest rate of allergic sensitization.

This study has some limitations. First, the sample size of the GWAS used to construct the IgE_PRS (*n* = 1,287) was modest. Larger GWAS would likely increase statistical power and enable the identification of additional IgE-associated loci^33^. Second, the methods used to assess allergic sensitization varied between the non-asthmatic controls and patients with asthma. Although multiplex assays were used in the control group (View Allergy 39 or MAST 26), the asthma cohort included both multiplex and single-antigen RAST. Given the known discrepancies in sensitivity and specificity across testing platforms^34^, these differences might have affected the comparability of the sensitization data. Third, the IgE_PRS developed in this study was not validated in an independent external cohort. Although we used the LOGO PRS approach to mitigate overfitting, external validation was essential to confirm the generalizability of our findings. Fourth, this study was limited to a Japanese population. Given the differences in allele frequencies across ethnicities, the applicability of the IgE_PRS to non-Japanese populations remains uncertain. Further studies in diverse populations are warranted. Fifth, GWAS performed for comparing Cluster 1 with non-asthmatic controls and the combined group of Clusters 2–4 was exploratory in nature and should be interpreted with caution. The sample size in Cluster 1 was relatively small for genome-wide analyses, resulting in reduced statistical power and a higher likelihood of unstable associations. Studies with larger sample sizes with independent replication cohorts and more appropriate comparator groups are needed to validate these findings. Sixth, we assessed additional external factors that could contribute to asthma heterogeneity—including family history, family structure, residential area, income, and comorbidities such as gastroesophageal reflux disease, sleep apnea syndrome, and chronic rhinosinusitis. However, these data were not sufficiently available in our dataset and therefore could not be incorporated into the analysis. This limitation should be taken into account when interpreting the identified cluster structure. Finally, although we used the IgE_PRS as a genetic instrument to explore potential causal relationships, we did not perform formal sensitivity analyses to assess horizontal pleiotropy^35^. Thus, we cannot exclude the possibility that variants influence asthma risk through pathways other than total IgE levels, which may limit the causal interpretation of our findings.

## Conclusion

In this study, we constructed a PRS for total IgE levels and used it as a genetic instrument to identify a subpopulation of adult-onset-predominant asthma with T2 characteristics who had a genetic predisposition associated with elevated total IgE levels. Furthermore, in a 10-year retrospective cohort study of non-asthmatic controls, we observed that this phenotype accounted for a certain proportion of newly developed adult-onset asthma. These results provide new insights into the heterogeneity of asthma and suggest the existence of genetically driven IgE-related endotypes. Future studies should further characterize this endotype and explore its potential utility as a guide for personalized treatment.

## Methods

### Study participants

This study included non-asthmatic controls who underwent periodic health checkups at the Total Health Evaluation Center Tsukuba and were not diagnosed with asthma or COPD at the time of registration and 829 patients with asthma who were attending Tsukuba University Hospital or its affiliated hospitals. Although the cohort was fundamentally the same as that used in our previous study^36^, the increase in sample size reflected the inclusion of 46 patients with asthma who were newly genotyped for the present analysis. Asthma was diagnosed based on the presence of at least two recurrent symptoms, including cough, wheezing, and shortness of breath, along with reversible airway obstruction or airway hyperresponsiveness to methacholine^37^. Questionnaires were administered to non-asthmatic controls and patients with asthma to collect information on age, sex, smoking history, and history of allergic diseases, including atopic dermatitis and allergic rhinitis. Patients with asthma were also interviewed regarding their age at asthma onset. To improve accuracy, they were asked in detail about the presence of coughing, wheezing, and shortness of breath during childhood and adolescence. In addition, serum samples were collected to measure total IgE levels using a chemiluminescent enzyme immunoassay-based analyzer. Furthermore, non-asthmatic controls diagnosed with asthma or COPD for the first time during the 10-year observation period after registration were excluded. Data of patients with asthma with missing data on total IgE levels were excluded from the analysis.

### Genotyping and quality control

Genomic DNA was extracted from the peripheral blood samples using an automated DNA extraction system (Quick Gene-610 L; Fujifilm, Tokyo, Japan). In a previous study conducted in our laboratory, GWASs were conducted using the Illumina HumanHap550v3/610-Quad BeadChip (Illumina, San Diego, CA, USA) in both healthy individuals and patients with asthma^38^. In the current study, we reanalyzed genetic data using the Infinium Asian Screening Array-24 v1.0 BeadChip. The whole-genome sequencing data used to design the Infinium Asian Screening Array included samples from the East and Southeast Asian populations^39^. This provides accurate information on SNP for the Japanese population.

To avoid overestimation of the PRS owing to the presence of genetically related individuals, first- and second-degree relatives within the non-asthmatic control group were identified and removed using PLINK version 1.90. Additionally, controls with extremely high or low heterozygosity – defined as values beyond ± 3 SDs from the cohort mean – were excluded, as these patterns may indicate related individuals within the cohort or poor DNA quality^40^. Individuals with > 10% missing genotypes across all SNPs were excluded from the analysis. Regarding SNP-level quality control, SNPs with a genotype call rate of < 0.99, a minor allele frequency of < 0.01, or a Hardy–Weinberg equilibrium p-value of < 1.0 × 10⁻⁶ were excluded. These thresholds were based on the standard GWAS quality control recommendations^41^. In the asthma cohort, data of individuals who showed very high pairwise genetic relatedness were also identified and excluded from subsequent analyses.

### Clinical measurements

Serum samples were collected from non-asthmatic control participants and patients with asthma to measure total IgE levels and perform allergen-specific IgE antibody testing. This was performed using one of the following methods: the MAST26 panel^42^, View Allergy 39 panel^43^, or the RAST test. In the MAST26 panel, sensitization to inhalant allergens was defined as a positive result for at least one of the following 14 inhalant allergens: mites, house dust, cats, dogs, pollen, and fungi. In the View Allergy 39 panel, sensitization was defined as a positive result for at least one of the 18 inhalant allergens, including mites, house dust, cats, dogs, pollen, fungi, moths, cockroaches, and latex. In the RAST test, sensitization was defined as a positive result for at least one of the 14 inhalant allergens, including mites, house dust, cats, dogs, pollen, and fungi^44^. Peripheral blood eosinophil counts were assessed in patients with asthma. Pulmonary function tests were performed in both non-asthmatic controls and patients with asthma.

### Calculation of IgE_PRS using the leave-one-group-out meta-analysis method

To identify the genetic determinants of total IgE levels independent of asthma and atopic dermatitis, we conducted a GWAS restricted to participants without these conditions. Individuals with asthma or atopic dermatitis were excluded to minimize confounding factors associated with disease-associated increases in serum total IgE levels^45^, as both conditions elevate IgE levels. Based on GWAS results, we constructed a PRS for total IgE levels (IgE_PRS), which was subsequently applied to a cohort of patients with asthma.

The IgE_PRS was calculated by dividing the dataset into two groups: one for conducting GWASs, which served as the base group, and the other for deriving the PRS, which served as the target group. We applied the LOGO meta-analysis approach^46^ in which the cohort was randomly divided into five non-overlapping subgroups. In each iteration, GWAS for total IgE levels was performed using four subgroups; the resulting SNP effect sizes were used to calculate PRSs in the remaining subgroup. The same set of SNPs was then used to calculate PRSs in all participants in that iteration. This process was repeated five times, generating five PRS estimates per individual. Moreover, the five resulting PRS estimates were subsequently meta-analyzed to generate the final IgE_PRS.

The detailed procedure is described below.

Initially, the control participants were randomly divided into five subgroups using the sample() function in R version 4.3.2 (https://www.r-project.org/). From these five subgroups, five different base cohorts, each comprising four subgroups, were created by excluding one subgroup in turn. GWAS for total IgE levels was conducted using PLINK version 1.90 in each of the five base cohorts. Covariates included age, sex, smoking status, genetic principal components, and presence or absence of allergen sensitization.

For each of the five combinations, the GWAS results from the base cohort were used to calculate the PRS in the corresponding target subgroup using PRSice-2^47^ (PRSice-2). The clumping and thresholding method was applied to select SNPs that contributed to the PRS. Clumping excluded SNPs in high linkage disequilibrium with the index SNP, retaining only the most significantly associated SNPs. Thresholding removes SNPs with p-values above a specified threshold, thereby reducing noise from weakly associated variants^48^.

The PRS was calculated using the following formula:

$${\mathrm{PR}}{{\mathrm{S}}_j}={\mathrm{(1}}/{{\mathrm{M}}_j}{\mathrm{)}} \times {\Sigma _i}({{\mathrm{S}}_i} \times {{\mathrm{G}}_{ij}})$$

where S_*i*_ is the summary statistic for the effective allele at SNP*i* and G_*ij*_ is the number of effective alleles (0, 1, or 2) observed for individual *j* at SNP_*i*_.

M_*j*_ is the number of SNPs included in the PRS calculation for individual *j*.

PRSice-2 enables the visualization of the association between a trait and PRSs calculated under various p-value thresholds, allowing for the determination of the threshold that yields the strongest correlation^47^.

Using this optimized threshold, the same set of SNPs was then used for calculating PRSs in all participants in each iteration. The five resulting PRSs were subsequently combined to obtain a single unified IgE_PRS per individual, using inverse-variance weighting based on the standard errors (SEs) of the regression coefficients representing the PRS–IgE association in each iteration. This inverse-variance approach followed the general principle of a fixed-effect meta-analysis, in which estimates from multiple models are combined according to their precision^49^ (Chap. 10: Analyzing data and performing meta-analyses | Cochrane), and provides a statistically valid and efficient summary of the overall PRS–IgE relationship while enhancing estimation stability and minimizing potential overfitting.

The final meta-analytic IgE_PRS was calculated as follows:

$${\mathrm{IgE}}\_{\mathrm{PRS}}=\Sigma ({\mathrm{PRS}}i \times {\mathrm{1}}/{\mathrm{SE}}{i^2})/\Sigma ({\mathrm{1}}/{\mathrm{SE}}{i^2})$$

where PRS*i* is the PRS calculated using one of the five PRS models and SE*i* is the standard error (*i* = 1–5).

The same formula for IgE_PRS calculation was applied to patients with asthma. The calculations were performed using PLINK version 1.90. Subsequently, as with the controls, the five IgE_PRS values were meta-analyzed using the inverse-variance method in R version 4.3.2. For ease of interpretation, the IgE_PRS values were standardized (z-scored) using the mean and standard deviation of the non-asthmatic control participants included in the present analysis.

### Statistical analyses

A comparison of baseline characteristics between non-asthmatic controls and asthmatic patients was conducted. Demographic and clinical variables, including age, sex, BMI, smoking status, allergic sensitization, and lung function were compared between the two groups using appropriate statistical tests. We performed linear regression analyses, adjusting for age, sex, smoking status, and allergen sensitization, to compare the total IgE levels and IgE_PRS between non-asthmatic controls and patients with asthma. Furthermore, the participants were stratified into four groups based on their asthma status and allergic sensitization.

To identify clusters characterized by high IgE_PRS and elevated total IgE levels, cluster analysis was performed using IgE_PRS, total IgE levels, age at asthma onset, and pFEV₁ as input variables. Age at asthma onset and pFEV₁ were selected because they were strong discriminators among adult patients with asthma in our previous study^37^. We used IBM SPSS Two-Step Cluster method with automatic determination of the number of clusters using the BIC, and adopted the number of clusters automatically selected by the algorithm. Two-Step Cluster first builds a cluster-features (CF) tree (preclustering) and then performs clustering on the preclusters. The optimal number of clusters is determined by minimizing the BIC, which balances between goodness-of-fit and model simplicity.

Comparisons of IgE_PRS (among non-asthmatic controls and the four asthma clusters) and clinical characteristics (among the four asthma clusters) were performed according to the distributional properties of each variable. Normality was assessed by the Shapiro–Wilk test, while homogeneity of variances was assessed by Levene’s test. Since most variables violated these assumptions, comparisons of IgE_PRS and clinical characteristics were performed using the Kruskal–Wallis test, followed by Dunn-Bonferroni-adjusted pairwise post-hoc analyses.

All statistical analyses were performed using IBM SPSS Statistics version 29.0.0.0.

### Functional mapping and annotation GWAS analysis

To further investigate the genetic background underlying the pathophysiology of Cluster 1, we performed GWAS twice using two separate control groups: non-asthmatic individuals used for IgE_PRS construction and the combined group of Clusters 2–4. GWAS was performed using PLINK version 1.90, without adjusting for covariates. To identify genes potentially associated with the pathophysiology of Cluster 1, we used FUMA GWAS (https://fuma.ctglab.nl/)^50^, which is a web-based platform that integrates multiple biological databases to functionally annotate GWAS results, allowing for the identification of disease-associated SNPs and genes.

We analyzed the lead SNPs and their surrounding genomic regions to identify candidate genes potentially associated with Cluster 1. We set a suggestive significance threshold of *p* < 1.0 × 10⁻⁵ for the selection of lead SNPs. Based on the identified SNPs, we used FUMA GWAS to perform gene mapping using three approaches: positional mapping, quantitative expression trait loci (eQTL) mapping, and chromatin interaction mapping.

### A 10-year retrospective cohort study on asthma development

To investigate the characteristics of adult-onset asthma, we conducted a longitudinal, retrospective, observational cohort study over 10 years involving 1,532 non-asthmatic controls. During the observation period, participants who developed asthma or COPD were identified^51^.

Based on retrospective cohort data, we evaluated whether patients newly diagnosed with asthma could be classified into one of the four clusters identified in the previous section. To classify new participants with asthma according to the established clustering framework, we applied a nearest-centroid assignment method^52^, which assigns each participant to the cluster whose centroid is closest in Euclidean distance.

We constructed a model using the original four asthma clusters as the dependent variable and age at asthma onset, pFEV₁, total IgE levels, and IgE_PRS as the independent variables. The constructed model was then applied to patients with newly developed asthma to determine the cluster assignment in each individual. All analyses were performed using R version 4.3.2.

## Supplementary Information

Below is the link to the electronic supplementary material.

Supplementary Material 1

## Data Availability

The GWAS genotype data of the non-asthmatic controls and asthma patients collected by our group at the University of Tsukuba cannot be deposited in a public repository, since no consent was obtained for deposition ***. However, these data are available upon request (contact: nhizawa@md.tsukuba.ac.jp) for use in research on inflammatory lung diseases.***.
