# Supplementary material for: Genetic predisposition to elevated total immunoglobulin E levels defines a distinct adult-onset-predominant asthma phenotype
Source: Sci Rep. 2026 Jan 29;16:6597. doi: 10.1038/s41598-026-37679-5 (PMC12913924; doi:10.1038/s41598-026-37679-5)
Supplement: Supplementary file 1 — Supplementary Material 1 [file 41598_2026_37679_MOESM1_ESM.pdf]

Supplementary Information

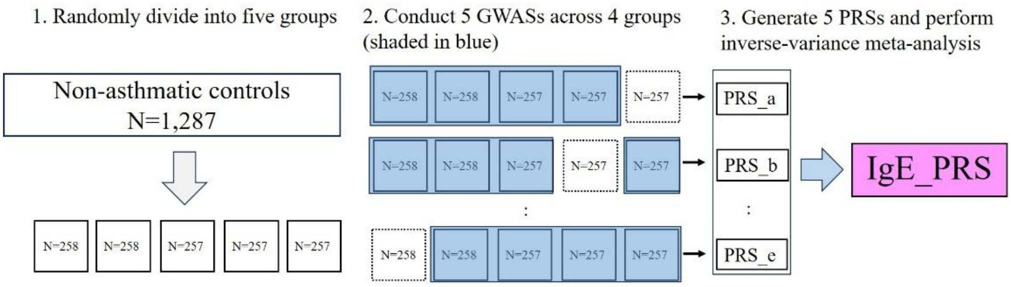

Supplementary Figure S1. Flowchart of immunoglobulin E_polygenic risk score (IgE_PRS) calculation in non-asthmatic controls using the leave-one-group-out PRS method.

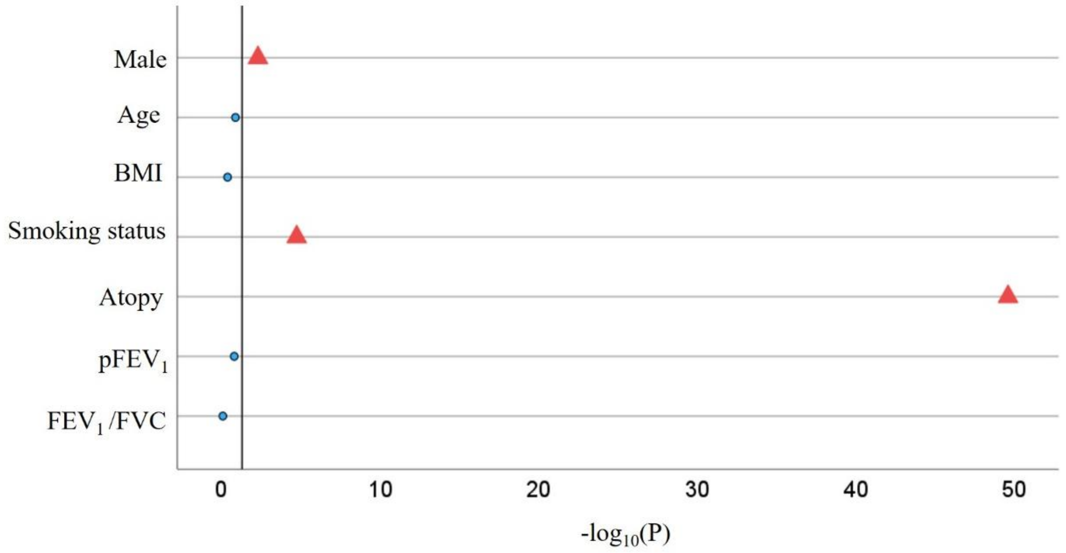

Supplementary Figure S2. Correlation between clinical parameters and total immunoglobulin E (IgE) levels in 1,287 non-asthmatic controls.

Linear regression analysis was performed for each clinical parameter (sex, age, BMI, smoking status, allergic sensitization, $pFEV_1$, FEV_1/FVC, and allergic rhinitis), using all other parameters as covariates. The solid line represents the significance threshold (p = 0.05, $-\log_{10}[P] = 1.30$). Red triangles indicate a positive correlation with total IgE levels, whereas blue circles indicate no significant correlation.

The figure was created using IBM SPSS Statistics version 29.0.0.0.

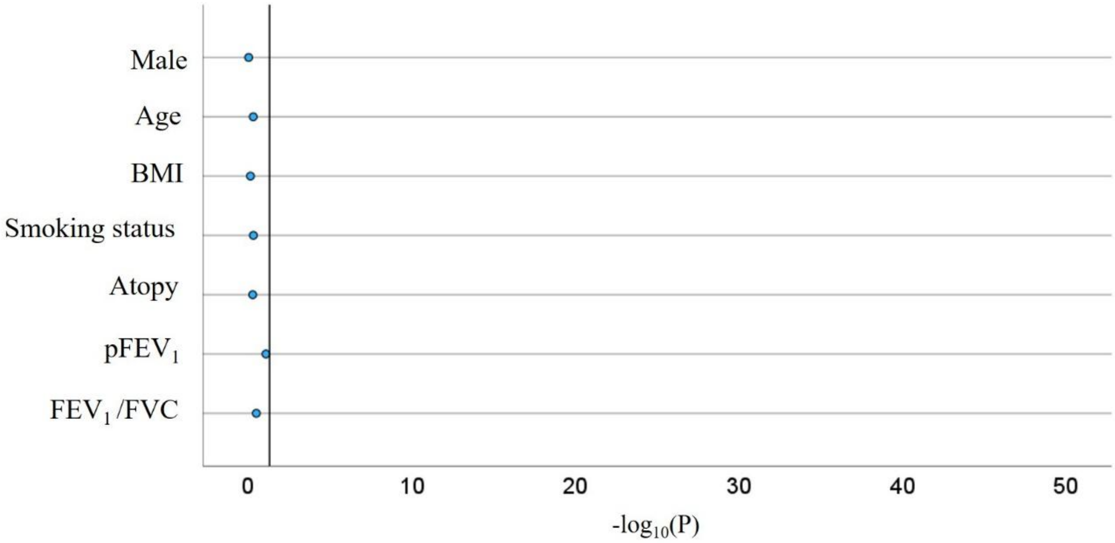

Supplementary Figure S3. Correlation between clinical parameters and immunoglobulin E_polygenic risk score in 1,287 non-asthmatic controls.

Linear regression analysis was performed for each clinical parameter (sex, age, BMI, smoking status, allergic sensitization, $pFEV_1$, FEV_1/FVC, and allergic rhinitis), using all other parameters as covariates. The solid line represents the significance threshold (p = 0.05, $-\log_{10}[P] = 1.30$). Blue circles indicate no significant correlations.

The figure was created using IBM SPSS Statistics version 29.0.0.0.

Supplementary Figure S4. Changes in Bayesian Information Criterion (BIC) and silhouette index across models with different numbers of clusters in the Two-Step cluster analysis.

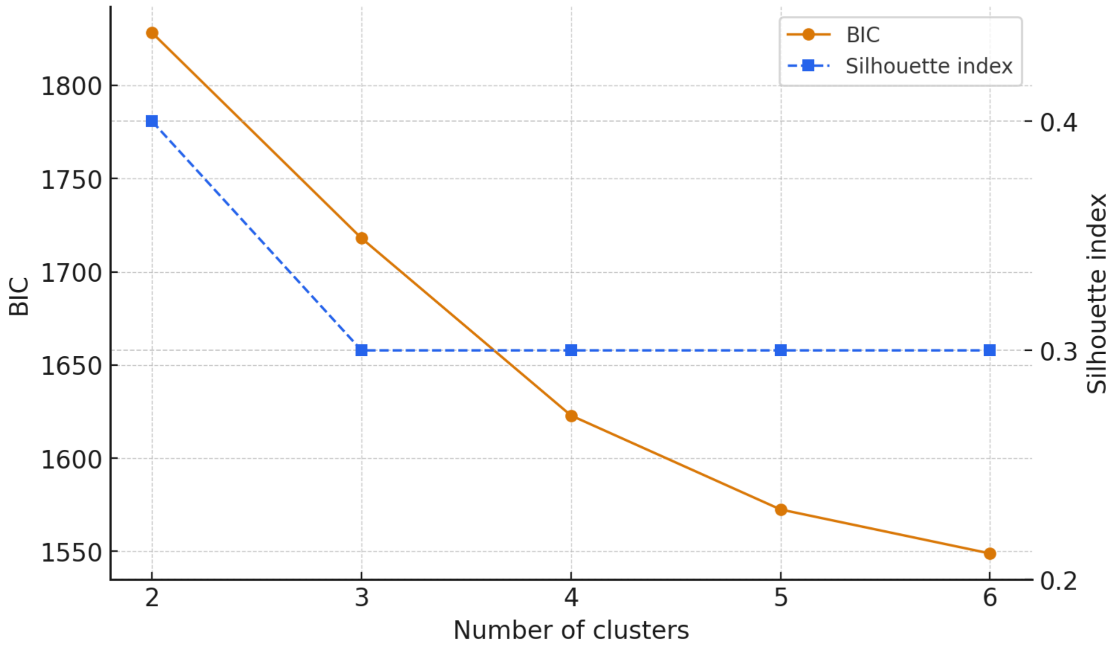

The automatically generated Two-Step models show a clear plateau in BIC reduction after four clusters, supporting the selection of a four-cluster solution as optimal.

Supplementary Table S1. IgE_PRS of the four asthma clusters and non-asthmatic controls (NACs).

	Cluster 1	Cluster 2	Cluster 3	Cluster 4	NACs	P*
Number of subjects	199	157	178	173	1,287	
IgE_PRS Z score (SD)	0.80 (0.46)	0.20 (0.75)	-0.41 (0.57)	-0.45 (0.46)	0.00 (1.00)	$< 1.0 \times 10^{-15}$

*IgE_PRS Z score was compared using the Kruskal-Wallis test.

Supplementary Table S2. Bonferroni-adjusted post-hoc comparisons of IgE_PRS across the four asthma clusters and non-asthmatic controls (NACs).

Comparison	Adjusted p-value
Cluster 1 vs Cluster 2	4.6×10^{-12}
Cluster 1 vs NACs	$< 1.0 \times 10^{-15}$
Cluster 1 vs Cluster 3	$< 1.0 \times 10^{-15}$
Cluster 1 vs Cluster 4	$< 1.0 \times 10^{-15}$
Cluster 2 vs NACs	0.032
Cluster 2 vs Cluster 3	2.3×10^{-10}
Cluster 2 vs Cluster 4	1.2×10^{-12}
Cluster 3 vs NACs	1.6×10^{-8}
Cluster 3 vs Cluster 4	1.000
Cluster 4 vs NACs	2.4×10^{-11}

Supplementary Table S3. Bonferroni-adjusted post-hoc comparisons of clinical parameters across the four clusters.

Comparison	Age	Age at asthma onset	BMI	Total IgE levels (log)	Blood eosinophil count (log)	$pFEV_1$	FEV_1/FVC
Cluster 1 vs Cluster 2	$< 1.0 \times 10^{-15}$	$< 1.0 \times 10^{-15}$	1.000	6.0×10^{-8}	0.974	0.025	1.000
Cluster 1 vs Cluster 3	0.598	1.000	1.000	7.9×10^{-9}	1.000	$< 1.0 \times 10^{-15}$	6.1×10^{-10}
Cluster 1 vs Cluster 4	0.526	0.012	1.000	5.9×10^{-4}	1.000	1.1×10^{-11}	0.0071
Cluster 2 vs Cluster 3	$< 1.0 \times 10^{-15}$	$< 1.0 \times 10^{-15}$	1.000	1.000	1.000	1.9×10^{-9}	3.5×10^{-9}
Cluster 2 vs Cluster 4	$< 1.0 \times 10^{-15}$	$< 1.0 \times 10^{-15}$	1.000	$< 1.0 \times 10^{-15}$	0.184	$< 1.0 \times 10^{-15}$	0.018
Cluster 3 vs Cluster 4	1.000	0.0099	1.000	$< 1.0 \times 10^{-15}$	0.274	$< 1.0 \times 10^{-15}$	$< 1.0 \times 10^{-15}$

All values represent Bonferroni-adjusted p-values from post-hoc pairwise comparisons.

Supplementary Table S4. List of genes mapped to significant single nucleotide polymorphisms from Cluster 1 genome-wide association study (GWAS) versus non-asthmatic controls.

Ensembl ID	Gene symbol	Ensembl ID	Gene symbol
ENSG00000137312	*FLOT1*	ENSG00000196126	*HLA-DRB1*
ENSG00000196260	*SFTA2*	ENSG00000223534	*HLA-DQB1-AS1*
ENSG00000228789	*HCG22*	ENSG00000263649	*MIR3135B*
ENSG00000204540	*PSORS1C1*	ENSG00000243753	*HLA-L*
ENSG00000204538	*PSORS1C2*	ENSG00000225851	*HLA-S*
ENSG00000204536	*CCHCR1*	ENSG00000272221	*MICA-AS1*
ENSG00000204531	*POU5F1*	ENSG00000204301	*NOTCH4*
ENSG00000204528	*PSORS1C3*	ENSG00000196301	*HLA-DRB9*
ENSG00000271581	*HCG4*	ENSG00000198502	*HLA-DRB5*
ENSG00000204520	*MICA*	ENSG00000196735	*HLA-DQA1*
ENSG00000222895	*RNU6-1133P*	ENSG00000179344	*HLA-DQB1*
ENSG00000204516	*MICB*	ENSG00000232629	*HLA-DQB2*
ENSG00000204542	*C6orf15*	ENSG00000133401	*PDZD2*
ENSG00000204539	*CDSN*	ENSG00000229391	*HLA-DRB6*
ENSG00000137411	*VARS2*	ENSG00000206344	*HCG27*
ENSG00000137310	*TCF19*	ENSG00000249774	*TOMM7P*
ENSG00000272501	*HCG27*	ENSG00000266243	*MIR4279*
ENSG00000204525	*HLA-C*	ENSG00000228022	*HCG20*
ENSG00000204356	*NELFE*	ENSG00000204580	*DDR1*
ENSG00000244731	*C4A*	ENSG00000234745	*HLA-B*
ENSG00000241106	*HLA-DOB*	ENSG00000199332	*Y_RNA*
ENSG00000226030	*HLA-DQB3*	ENSG00000204438	*GPANK1*
ENSG00000235040	*MTCO3P1*	ENSG00000213722	*DDAH2*
ENSG00000237541	*HLA-DQA2*	ENSG00000243649	*CFB*
ENSG00000204267	*TAP2*	ENSG00000204348	*DXO*
ENSG00000204314	*PRRT1*	ENSG00000250535	*STK19P*

Genes were mapped using positional, eQTL, or chromatin interaction mapping via Functional Mapping and Annotation GWAS. fifty-two genes were identified.

Supplementary Table S5. List of genes mapped to significant single nucleotide polymorphisms from Cluster 1 genome-wide association study (GWAS) versus the combined group of Clusters 2–4.

Ensembl ID	Gene symbol	Ensembl ID	Gene symbol
ENSG00000252650	*RNA5SP75*	ENSG00000204531	*POU5F1*
ENSG00000251861	*SCARNA20*	ENSG00000204528	*PSORS1C3*
ENSG00000243753	*HLA-L*	ENSG00000272501	*HCG27*
ENSG00000137312	*FLOT1*	ENSG00000204525	*HLA-C*
ENSG00000204580	*DDR1*	ENSG00000271581	*HCG4*
ENSG00000137411	*VARS2*	ENSG00000225851	*HLA-S*
ENSG00000196260	*SFTA2*	ENSG00000272221	*MICA-AS1*
ENSG00000228789	*HCG22*	ENSG00000199332	*Y_RNA*
ENSG00000222895	*RNU6-1133P*	ENSG00000204520	*MICA*
ENSG00000204542	*C6orf15*	ENSG00000204516	*MICB*
ENSG00000204540	*PSORS1C1*	ENSG00000204438	*GPANK1*
ENSG00000204539	*CDSN*	ENSG00000244731	*C4A*
ENSG00000204538	*PSORS1C2*	ENSG00000227438	*AP001471.1*
ENSG00000204536	*CCHCR1*	ENSG00000142173	*COL6A2*
ENSG00000137310	*TCF19*		

Genes were mapped using positional, eQTL, or chromatin interaction mapping via Functional Mapping and Annotation GWAS. Twenty-nine genes were identified.

Supplementary Table S6. Clusters of newly diagnosed asthma and their characteristics.

	Cluster 1	Cluster 3	Cluster 4
Number of subjects	25	8	46
Sex (female, %)	11 (44.0)	2 (25.0)	36 (78.3)
Age, y (range)	49.1 (31-72)	46.1 (37-58)	50.7 (31-72)
Age at asthma onset (range)	54.7 (39-78)	51.3 (45-60)	57.0 (40-81)
BMI (SD)	23.1 (3.0)	25.6 (3.0)	23.3 (3.1)
Smoking pack-years (%)			
0	16 (64.0)	4 (50.0)	39 (84.8)
0-10	4 (16.0)	1 (12.5)	2 (4.3)
>10	5 (20.0)	3 (37.5)	5 (10.9)
Atopy (%)	17 (68.0)	6 (75.0)	21 (45.7)
Atopic dermatitis (%)	1 (4.0)	0 (0.0)	3 (6.5)
Total IgE levels (log, SD)	1.93 (0.43)	2.73 (0.60)	1.61 (0.46)
IgE_PRS Z score (SD)	0.87 (0.48)	-0.61 (0.47)	-0.42 (0.57)
FEV_1 (%, SD)	90.0 (11.2)	77.2 (6.8)	95.6 (11.9)
FEV_1 /FVC (%, SD)	80.5 (5.7)	81.8 (5.0)	83.3 (5.0)

Of the 79 newly diagnosed asthma patients, two patients in Cluster 4 had missing pulmonary function data.
